# Comparison between Communicated and Calculated Exposure Estimates Obtained through Three Modeling Tools

**DOI:** 10.3390/ijerph17114175

**Published:** 2020-06-11

**Authors:** Andrea Spinazzè, Francesca Borghi, Daniele Magni, Costanza Rovida, Monica Locatelli, Andrea Cattaneo, Domenico Maria Cavallo

**Affiliations:** 1 Dipartimento di Scienza e Alta Tecnologia, Università degli Studi dell’Insubria, Via Valleggio 11, 22100 Como, Italy; dmagni@studenti.uninsubria.it (D.M.); andrea.cattaneo@uninsubria.it (A.C.); domenico.cavallo@uninsubria.it (D.M.C.); 2 TEAM mastery S.r.l. Via Ferrari 14, 22100 Como, Italy; costanzarovida@team-mastery.eu (C.R.); monicalocatelli@team-mastery.eu (M.L.)

**Keywords:** occupational exposure assessment, advanced REACH tool (ART), ECETOC TRA, STOFFENMANAGER^®^, scaling, exposure scenario, risk characterization ratio, occupational exposure models, REACH

## Abstract

This study aims to evaluate the risk assessment approach of the REACH legislation in industrial chemical departments with a focus on the use of three models to calculate exposures, and discuss those factors that can determine a bias between the estimated exposure (and therefore the expected risk) in the extended safety data sheets (e-SDS) and the expected exposure for the actual scenario. To purse this goal, the exposure estimates and risk characterization ratios (RCRs) of registered exposure scenarios (ES; “communicated exposure” and “communicated RCR”) were compared with the exposure estimates and the corresponding RCRs calculated for the actual, observed ES, using recommended tools for the evaluation of exposure assessment and in particular the following tools: (i) the European Centre for Ecotoxicology and Toxicology of Chemicals Targeted Risk Assessment v.3.1 (ECETOC TRA), (ii) STOFFENMANAGER^®^ v.8.0 and (iii) the Advanced REACH Tool (ART). We evaluated 49 scenarios in three companies handling chemicals. Risk characterization ratios (RCRs) were calculated by dividing estimated exposures by derived no-effect levels (DNELs). Although the calculated exposure and RCRs generally were lower than communicated, the correlation between communicated and calculated exposures and RCRs was generally poor, indicating that the generic registered scenarios do not reflect actual working, exposure and risk conditions. Further, some observed scenarios resulted in calculated exposure values and RCR higher than those communicated through chemicals’ e-SDSs; thus ‘false safe’ scenarios (calculated RCRs > 1) were also observed. Overall, the obtained evidences contribute to doubt about whether the risk assessment should be performed using generic (communicated by suppliers) ES with insufficient detail of the specific scenario at all companies. Contrariwise, evidences suggested that it would be safer for downstream users to perform scenario-specific evaluations, by means of proper scaling approach, to achieve more representative estimates of chemical risk.

## 1. Introduction

### 1.1. Background

Since the REACH regulation (EC 1907/2006) was introduced, the quantitative occupational exposure assessment to chemicals in both small-medium and large enterprises became a dominant topic in the field of exposure sciences and occupational hygiene, in relation to the high necessity of risk assessments. As defined by the European Chemical Agency (ECHA), it is possible to perform the occupational exposure assessment to chemicals by means of mathematical models, as an alternative or to complement environmental monitoring [1]. Thus, a constant development of exposure assessment models occurred [2], also in relation to the high number of chemicals present on the European and international market and the even higher number of possible occupational exposure scenarios. The assessment workflow outlined by ECHA suggests a tiered, step by step pathway [1] to obtain quantitative exposure estimates and risk characterization ratios (RCRs) for each relevant exposure route, type of effects, as well as combined RCRs (i.e., for dermal and inhalation exposure). The RCR for a certain exposure route and type of effect is calculated as the ratio between the exposure to a chemical obtained though model estimation and the corresponding derived no-effect level (DNEL) for that chemical. To obtain a controlled risk it is necessary to verify that the RCR is less than 1 (i.e., estimated exposure < DNEL). The provisions that downstream users must follow to obtain a controlled risk are communicated through the extended safety data sheets (e-SDSs) of chemicals [3]. The e-SDSs consists of SDSs (safety data sheets), a technical information documents on substances and mixtures, and their exposure scenarios (ES) which describe the permitted uses, operating conditions (OCs) and risk management measures (RMMs) to be respected for each activity performed within and highlighted by process categories (PROCs). To guarantee effective protection during exposure to chemical substances, it is essential to fulfill the instructions given in the ES [3]. For each of the identified uses in the lifecycle of a substance, the operational conditions and risk management measures ensuring control of risk must be determined. This set of information is called an exposure scenario (ES). An exposure scenario usually covers several contributing tasks/activities within the use. It should be noted that when the ES developed by the manufacturer or importer is transmitted in the supply chain (through the transmission of the e-SDSs) to the downstream users, this latter must determine if the OCs and the RMMs of the actual scenario are in accordance with the specifications given in the ES. If there are any differences in the OCs and RMMs, then the user is required to verify whether also in these different conditions (i.e., in the actual scenario) the use of the substance is safe (i.e., if the RCR value is below 1) [4]. A calculated RCR higher than 1, indicates the need to apply improved RMMs. In this context, the “scaling” process refers to any operative process that allows one to recalculate the risk depending on the OCs and RMMs associated with the use of the chemical. The scaling can be used only for the parameters specified by the supplier, and only according to the measurement tools or models that have been used for the RCR calculation in the ES. The use of the scaling is not allowed if the adjustment of a crucial factor produces different exposure routes, or exposure affects different target groups or if the duration and/or frequency of exposure changes significantly, resulting in a different kind of exposure (for example, acute exposure rather than chronic exposure). Thus, the mechanism of the scaling consists in calculating the RCR of the actual scenario by changing the OC and RMM variables provided by the supplier in the ES and introducing the user’s specific OC and RMM. The application of mathematical models for scaling requires a good knowledge about issues associated with exposure, as well as proper understanding of all OC and RMM modes [1,4,5].

### 1.2. Problem Statement

The risk assessment carried out for the preparation of the exposure scenarios with a forecast approach, and the subsequent verification of correspondence of the real ES with respect to the e-SDS—or any calculation to adjust the real ES to that e-SDS (scaling)—are based on the use of advanced mathematical models to estimate exposure. Nevertheless, recent studies have revealed the need to revise these exposure models and to evaluate their reliability in terms of accuracy, precision and robustness [2,6]. The reliability of the exposure estimates appears particularly relevant when these models have been used to carry out the exposure estimates regarding exposure scenarios of e-SDS, due to the risk of accepting false safe scenarios. These refer to situations in which risk assessment for a generic ES based on models were deemed safe in the e-SDS (i.e., estimated exposure < DNEL; RCR < 1), but the exposure estimates for the actual ES showed potential unsafe situations (i.e., estimated exposure > DNEL; RCR > 1) [7]. Further, attention was already posed to the ESs characterization, i.e., the determination of PROCs, RMMs and OCs, which, if not properly performed (resulting in less precision and uncertainty), can lead to misinterpretation of exposure estimates; similarly, the possibility of obtaining significantly different data through the use of different models for the same ES (resulting in low accuracy of the exposure assessment) was documented [8,9,10,11,12]. Having pointed out this problem, more in-depth analysis was conducted with the aim of comparing the exposure estimates observed and the RCRs, calculated by different models, with those of the registered ESs [7], while other studies focused on the comparison between modeled RCRs and measured RCRs from exposure monitoring [13,14].

### 1.3. Objectives

This study aims to evaluate the risk assessment approach of the REACH legislation in industrial chemical departments with a focus on the use of three models to calculate exposures, and determine any bias between the estimated exposure (and therefore the expected risk) in the e-SDS and the expected exposure for the actual scenario. To purse this goal, the exposure estimates and RCRs of registered ES (“communicated exposure” and “communicated RCR”) were compared with the exposure estimates and the corresponding RCRs calculated for the actual, observed ESs, using recommended tools by ECHA for the evaluation of exposure assessment [1] and in particular the following tools: (i) the European Centre for Ecotoxicology and Toxicology of Chemicals Targeted Risk Assessment v.3.1 (ECETOC TRA, Brussels, Belgium), (ii) STOFFENMANAGER^®^ v.8.0 (www.stoffenmanager.com; Cosanta BV, Schiphol-Oost, The Netherlands) and (iii) the Advanced REACH Tool v.1.5 (ART, Zeist, The Netherlands; www.advancedreachtool.com).

## 2. Materials and Methods

### 2.1. Data Collection

The rationale of the study, derived from a previous similar study [7], required to obtain exposure estimates through models and their corresponding calculated RCRs derived from observed ESs and compare them with the corresponding communicated exposure estimates and RCRs. With this purpose, data concerning ESs registered in selected companies, located in Italy, were collected; subsequently inspections were carried out to describe the selected ESs as observed. Eight companies were contacted and made themselves available for the preliminary assessment of the exposure scenarios present in the company. Subsequently, having verified the unavailability of ESs in five companies, three companies joined the study and therefore the reconstruction phase of the actual ESs. Five suitable exposure cases were selected from each company for a total of five chemicals considered (Table 1).

In accordance with the representatives of the three enrolled companies, inspections were carried out to collect the data necessary for the exposure assessment. During each visit, the parameters needed to assess the observed ESs when the chemical of interest was used were recorded, with particular emphasis on information about how the substances were handled at the worksite. The parameters collected for each observed exposure scenario were subsequently translated into a spreadsheets database on the EXCEL™ software (Microsoft, Redmond, WA, USA).

### 2.2. Exposure Assessment—Modeling Tools

Three exposure assessment models were used to model the parameters observed during the visits to the three companies. In accordance with the ECHA Guidance on Information Requirements and Chemical Safety Assessment Chapter, R.14: Occupational Exposure Assessment [1] the exposure assessments were conducted using ECETOC TRA v.3.1 [15] STOFFENMANAGER^®^ v.8.0 [16,17] and ART v.1.5 [18,19]. 

ECETOC TRA is a generic model for inhalation and dermal occupational exposure, based on the descriptors used for processes categories (PROCs) defined under the REACH regulation [7,9]. The initial exposure estimates are derived from estimation and assessment of substance exposure (EASE) [20] but modified based on modifying factors [15]. STOFFENMANAGER^®^ is a web-based dermal and inhalation occupational exposure model [16], considered a more refined version of Tier 1 models [1,21,22]. The algorithm and general assumptions used in STOFFENMANAGER^®^ (versions 3.0–4.0) as well as the model’s calibration are widely descripted in scientific literature [16,17,23,24,25]; subsequent modifications to these earlier versions are listed on www.stoffenmanager.com. The 90th percentile outcomes are recommended for this model to ensure a conservative result [1,21]. ART is the most sophisticated and advanced tool for exposure modeling recommended under the REACH regulation [7]; the model is based on a source–receptor mechanistic model combined with an empirical part related to exposure database [18,22]. The 75th or the 90th percentile of estimates is recommended to be used as outcomes for this model.

### 2.3. Exposure Assessment—Exposure Modeling

The observed working conditions at the worksites were modeled using ECETOC TRA, STOFFENMANAGER^®^ and ART. These modeled scenarios are referred to as the operating conditions and risk management measures observed during the inspections carried out in the enrolled companies. The results of the calculations reflect respectively the 75th percentile for ECETOC TRA v.3.1 and the 90th percentile for ART v.1.5 and STOFFENMANAGER^®^ v.8.0. The chemical–physical properties used for exposure modeling are those reported in the SDSs. When these data were not available, they were collected from databases and online resources such as GESTIS Substance database (IFA, Berllin, Denmark; www.dguv.de/ifa/gestis-database), PubChem (PubChem, Bethesda, MD, USA https://pubchem.ncbi.nlm.nih.gov/) and ECHA’s Registered substances factsheets (ECHA, Helsinki, Finland; https://echa.europa.eu/search-for-chemicals). The concentrations, control measures, and distance between the emission source and the worker’s breathing zone can differ between the specific observed scenarios and the registered ESs, especially because the latter are usually generic. Hence, often, the number of observed scenarios and PROCs, RMMs and OCs are different than the registered ES.

### 2.4. Data Analysis

The communicated exposure situations (49) were generated from the assessments of 5 different chemicals (Table 1); the estimated exposure values were obtained by means of ECETOC TRA (version 2.3 for ES related to the used of morpholine and 2,4,6-trichloro-1,3,5-triazine; version 3.1 for ES related to other chemicals). When feasible, the occupational exposures of the observed scenarios were estimated and the corresponding RCRs calculated by dividing the modeled exposure by the DNEL presented in the ES based on the actual conditions of use. The communicated RCRs from the registered ES were then compared with the RCRs of the observed scenarios (observed RCRs). Not all the exposure situations communicated in the ES were observed during the audit carried out, as not all of these were characteristic of the activity carried out in the companies. The scenarios calculated with more advanced tools were compared to those received in the e-SDS, so the focus was on scenarios in which both a calculated and communicated exposure and/or RCR values were obtained. Statistical analyses were performed using SPSS Statistics 20.0 software package (IBM, Armonk, NY, USA). A *p*-value lower than 0.05 was considered as statistically significant for all tests. Descriptive statistics were calculated for both communicated and calculated exposure estimates, as well as for main information concerning the ES characteristics. The comparison of the communicated versus the calculated exposure estimates and RCRs (also by using different modeling tools) was carried out using different tests. First, differences between communicated and calculated exposure and RCRs were calculated and Wilcoxon test was used to identify statistically significant differences (*p* < 0.05). Then, precision evaluation was performed: this consists of the evaluation of uncertainty by means of uncertainty analysis and linear regression according to the indications summarized by Watson et al. [26].

Finally, the uncertainty between communicated and calculated exposures and RCRs was calculated following the guidance reported by the EC Working Group [27]; the uncertainty was calculated from the difference of measure according to Equation (1):

(1) $$u_{bs}^{2}=\frac{\sum_{i=1}^{n}{\left(y_{i,1}-y_{i,2\;}\right)}^{2}}{2n}$$

**Equation (1).** Uncertainty formula used in this study. *u*^2^*_bs_* represents the uncertainty; *y_i_*_,1_ and *y_i_*_,2_ represent the communicated (*y_i_*_,1_) and calculated (*y_i_*_,2_) estimated exposure or RCR; n represents the number of the total comparison considered in the analysis.

It should be noted that these two approaches are generally used to compare different methods of measurement (and not estimation methods) of exposure or concentrations of airborne pollutants [28,29]; however, in this study it was defined to adopt the same method, extending similar considerations also to the estimation methods. Linear regression was used to evaluate the level of agreement between the two methods and the reference method was considered as the independent variable while the method to be tested was the dependent variable. The communicated exposure estimates and RCRs were used as the independent variables and the calculated exposure estimates and RCRs were used as the dependent variable in the linear regression analysis. As reported by Watson et al. [26], equation parameters (R, slope and intercept) can be used as indicators of comparability and/or predictability between the two methods. In particular, the two methods can be classified as comparable and mutually predictable (i.e., the independent and dependent variables are considered interchangeable) if: (i) slope is equal to 1 ± 3 standard error (s.e.); (ii) intercept is equal to 0 ± 3 s.e. and (iii) R > 0.9. If R is > 0.9 but the slope and intercept criteria are not met, the investigated methods can be considered as comparable but only the dependent variable is predictable from the independent variable. Finally, methods with R < 0.9 are classified as not comparable. Finally, the numbers of all scenarios in which calculated exposures and RCRs were higher than those communicated and with calculated RCRs higher than 1 are summarized and displayed, to outline those situations in which a possible underestimation of exposure and of the risk were communicated.

## 3. Results

### 3.1. Scenario Characteristics

The data relating to the physical–chemical properties were collected, in 14 out of 20 cases, from online sources and databases (Table 2). The source from which the most data were collected is ECHA’s Registered Substances Factsheets (*N* = 9 cases). Pubchem and GESTIS in *N* = 4 and *N* = 1 cases, respectively. The data were available in e-SDS/SDS in 6 out of 20 cases. The chemical with more data available in the communicated scenarios is 2,4,6-trichloro-1,3,5-triazine with molecular weight (MW), vapor pressure (Vp) and the octanol–water partition coefficient (Log Kow) obtained from its own e-SDS/SDS. Data for 2,2′-iminodiethanol (90%) were collected only from online sources: MW and WS from Pubchem and VP, Log Kow from ECHA’s registered substances factsheets. DNEL and derived no-effect levels/derived minimal effect levels (DNEL/DMEL) were collected from e-SDS/SDS (*N* = 5) and ECHA’s registered substances factsheets (*N* = 6). Reference values for long-term inhalation exposure (systemic effects) and long-term dermal exposure for 3-isocyanatomethyl-3,5,5-trimethylcyclohexyl isocyanate respectively were not available. Long-term inhalation exposure (local effects) reference value for 2-[[(butylamino)carbonyl]oxy]ethyl acrylate was not available due to unexpected exposure (unknown and not identified hazard). Further, the long-term inhalation exposure (systemic effects) reference value of 2,4,6-trichloro-1,3,5-triazine was not available.

The most represented communicated scenario is “Use as intermediate or monomer” with *N* = 17 (34.7%) PROCs relating to this scenario description. The least represented was “Loading/filling” with N = 3 (6.1%) related PROCs. The PROC most represented among the communicated scenarios is 8b (transfer of substance or mixture (charging and discharging) at dedicated facilities; *N* = 6; 12.2%). PROCs 1 (chemical production or refinery in closed process without likelihood of exposure or processes with equivalent containment conditions), 3 (manufacture or formulation in the chemical industry in closed batch processes with occasional controlled exposure or processes with equivalent containment condition), 4 (chemical production where opportunity for exposure arises), 9 (transfer of substance or mixture into small containers (dedicated filling line, including weighing) and 15 (use as laboratory reagent) are equally represented (*N* = 4; 8.2%). PROC 6 (calendaring operations) represents 2% of the communicated PROCs (*N* = 1).

When considering the observed scenarios, discrepancies emerge between the communicated scenario descriptions and PROCs, and those that were actually observed (Table 3). Following the technical inspection in the investigated departments, *N* = 12 (38.7%) situations were interpreted as “Formulation and packaging/repackaging of substances and mixtures”; the “Industrial application of coatings, inks, adhesives and/or other formulations” scenario was represented by *N* = 1 (3.2%) PROC (Table 3). PROCs 8b, 9 and 15 were the most observed and they are equally represented with 10.20% (*N* = 5) each other. PROC 13 (Treatment of articles by dipping and pouring) was observed as it represented the 2% (*N* = 1) of Observed PROCs. Further, in *N* = 18 cases (36.7%), no correspondence in the investigated company was found for any of the communicated PROCs (PROC not relevant in the real scenario).

All the communicated exposure estimates were obtained by means of ECETOC TRA. The input variables required by the ECETOC TRA v.3.1 model are shown in Table 4. Additionally, in this case, there are appreciable differences between the characteristics of the communicated scenario and those of the actual scenario. In *N* = 6 (12.2%) cases, for the communicated scenarios the considered substance was a solid, of these *N* = 1 with low dustiness and *N* = 5 with medium dustiness. In the observed scenarios, the exposure situations referred to the solid chemical were *N* = 4 (12.9%), all with medium dustiness (100%). In *N* = 43 (87.8%) PROCs relating to the communicated scenarios the chemical was liquid, while in the observed scenarios this was defined only in *N* = 27 (87.10%) cases (Table 4). For the variable “Duration of the activity” in the communicated scenarios the value “>4 h” was characterizing *N* = 24 (49%) PROCs (*N* = 7–22.6% in the observed scenario). In the observed scenarios the value “15 min to 1 h” was representative of *N* = 16 (51.6%) PROCs. Activities with duration “<15 min” in the observed scenarios were not represented unlike those communicated where the parameter characterizes *N* = 4 (8.2%) PROCs, as well as the value “15 min to 1 h” (*N* = 4, 8.2%; Table 4). As reported in Table 4, in *N* = 33 (67.3%) of the communicated scenarios RPE (respiratory protective equipment) were not required; in fact, however, in all the observed scenarios RPE were in use: in *N* = 27 of the observed PROCs (87.1%) the efficiency of the RPE in use was 90% while the use of RPE with efficiency of 99% has never been observed, even if it was prescribed in *N* = 2 (4.1%) of the communicated scenario. In *N* = 29 (59.20%) PROCs the relative chemical was authorized for use with substance in preparation at concentrations “>25%” and in *N* = 20 (40.80%) with concentrations “5–25%”. In the observed scenarios for *N* = 4 (12.90%) PROCs, substance in preparation at concentrations “>25%” was observed while for *N* = 25 (80.60%) PROCs chemicals were used at concentrations at concentrations <1% (Table 4). The use of dermal PPE (personal protective equipment; gloves) in the communicated scenarios is defined for *N* = 32 (65.30%) PROCs where an assigned protection factor “APF = 10” was required; further, in *N* = 6 (12.20%) PROCs the use of gloves with “APF20” was requested. In the observed scenarios dermal PPE with “APF10” and “APF20”, were used in *N* = 27 (87.10%) and *N* = 4 (12.90%) PROCs, respectively. LEV (local exhaust ventilation) relating to dermal exposure in the communicated scenarios was requested in *N* = 27 (55.10%) PROCs. For the observed scenarios, LEV was used in *N* = 29 (93.50%) PROCs (Table 4).

### 3.2. Comparison of Exposure Estimates

As can be seen in Table 5 relating to the estimates of the communicated scenarios, the exposure values of inhalation exposure long-term (local effects) resulted in an average exposure (mean ± standard deviation) of 4.31 ± 6.94 mg/m^3^ for *N* = 23 valid exposure estimates; the corresponding RCRs were obtained for *N* = 13 valid exposure estimates with an average value of 4.21 × 10^−1^ ± 1.77 × 10^−1^. The lowest average exposure value was estimated for the inhalation exposure short-term (local effects), defined for *N* = 13 PROCs with 2.12 × 10^−2^ ± 8,94 × 10^−3^ mg/m^3^ and resulting in an average RCR of 4.69 × 10^−1^ ± 1.96 × 10^−1^.

Exposure estimates (75th percentile) calculated based on the observed scenario with the ECETOC TRA v.3.1 model, reported in Table 6, showed an average estimated exposure of 6.31 × 10^−1^ ± 9.58 mg/m^3^ for inhalation, short-term exposure (local effects) on *N* = 31 valid exposure estimates. The corresponding RCRs were obtained for *N* = 25 valid exposure estimates with an average value of 6.02 × 10^−1^ ± 1.07 × 10^−1^. A lower average exposure (7.72 × 10^−2^ ± 1.97 × 10^−1^ mg/m^3^) was obtained for *N* = 31 valid exposure estimates of inhalation exposure, long-term (systemic effects). The corresponding RCRs were obtained for *N* = 25 valid exposure estimates with an average value of 4.20 × 10^−2^ ± 1.33 × 10^−1^. The inhalation exposure long-term (local effects) was not evaluated in this case due to the model outputs did not consider this estimate.

Exposure estimates (90th percentile) and the corresponding RCR for both systemic and local effects. RCRs for inhalation long-term exposure were calculated with STOFFENMANAGER^®^ v.8.0. As shown in Table 7, the highest average calculated exposure estimates (2.45 ± 5.91 mg/m^3^) was obtained for inhalation exposure long-term (systemic effect) for a total of *N* = 31 valid exposure estimates; the corresponding RCRs showed an average value of 2.91 ± 1.09. Estimates (*N* = 25) for inhalation exposure long-term (local effects) resulted in an average exposure of 2.00 ± 6.45 mg/m^3^, thus resulting in an average RCR of 3.54 ± 1.21 × 10.

Exposure estimates (90th percentile) and the corresponding RCR for both systemic and local effects for inhalation long-term exposure were calculated with the ART v.1.5 model. As shown in Table 8, the highest average calculated exposure estimates (3.99 ± 1.47 × 10 mg/m^3^) was obtained for inhalation exposure long-term (local effect) for a total of *N* = 23 valid exposure estimates; the corresponding RCRs showed an average value of 1.19 × 10 ± 5.33 × 10. Estimates (*N* = 23) for inhalation exposure long-term (systemic effects) resulted in an average exposure of 3.67 ± 1.49 mg/m^3^, thus resulting in an average RCR of 1.14 ± 3.37 × 10.

### 3.3. Comparison between Communicated and Calculated Exposure Estimates

Following the calculation of the exposure estimates and the corresponding RCRs for the observed scenarios, a comparison was made between the communicated and the observed scenario for each model used. Differences were calculated between exposure and RCR estimates of communicated scenario and observed scenario (Table 9, Table 10 and Table 11). The Wilcoxon test was performed out to assess if the observed differences were statistically significant.

#### 3.3.1. Calculated Exposure Estimates—ECETOC TRA v.3.1

Table 9 shows a direct comparison between the reported exposure and RCR values and those calculated with ECETOC TRA. From the comparison it emerges that the average calculated dermal exposure and the corresponding RCR are lower than communicated dermal exposure, as well as for the estimates of inhalation, long-term exposure (systemic effects), for which calculated exposure values are on average lower than the communicated values of two orders of magnitude. Contrariwise, calculated estimates for inhalation, short-term exposure (local effects) and the corresponding RCRs showed average value higher than communicated values. The Wilcoxon test highlighted a statistical significance (*p* < 0.05) between communicated and calculated dermal exposures (*p* = 0.001) and the associated RCRs (*p* = 0.001), as well as for inhalation long-term exposures (*p* < 0.001) and the corresponding RCR for systemic effects (*p* = 0.001). For both inhalation short-term exposure (*p* = 0.068) and the corresponding RCRs for local effects (*p* = 0.068) the observed differences resulted to be not statistically significant.

#### 3.3.2. Calculate Exposure Estimates—STOFFENMANAGER^®^ v.8.0

Table 10 shows a direct comparison between the reported exposure and RCR values and those calculated with STOFFENMANAGER^®^ v.8.0. From the comparison it emerges that the median calculated exposures and the corresponding RCRs were lower than the communicated parameters. However, mean values outlined differences in the comparison between calculated and communicated exposures and RCRs, indicating how, on some occasions, the calculated value was much higher than the communicated value. The Wilcoxon test highlighted that the observed differences were not statistically significant (*p* > 0.05) between communicated and calculated inhalation long-term exposures (systemic effects: *p* = 0.940; local effects: *p* = 0.110) and the corresponding RCR for systemic effects (*p* = 0.078) and local effects (*p* = 0.068). For both inhalation short-term exposure (*p* = 0.068) and the corresponding RCRs for local effects (*p* = 0.068) the observed differences resulted to be not statistically significant.

#### 3.3.3. Calculate Exposure Estimates—ART v.1.5

Table 11 shows a direct comparison between the reported exposure and RCR values and those calculated with ART v.1.5. From the comparison it emerges that the average and the median calculated exposures and the corresponding RCRs were lower than the communicated parameters. However, the Wilcoxon test highlighted that the observed differences were not statistically significant (*p* > 0.05) between communicated and calculated when considering estimates of inhalation long-term exposures for local effects (*p* = 0.092) and the corresponding RCR (0.715). Contrariwise, calculated and communicated estimates of inhalation long-term exposure for systemic effects (*p* < 0.001) and the corresponding RCRs (*p* = 0.003) presented statistically significant differences.

#### 3.3.4. Liner Regression Analysis and Uncertainty Evaluation

As previously stated, linear regression analyses were carried out between calculated and communicated exposure estimates and RCR (the communicated exposure estimates and RCRs were used as the independent variables and the calculated exposure estimates and RCRs were used as the dependent variable in the linear regression analysis.), and regression parameters were used as indicators of precision, together with the calculation of the uncertainty. Results of this comparison are reported in Table 12, with the number of scenarios in which calculated exposures and RCRs were higher than those communicated (thus indicating a possible underestimation of exposure and of the risk were communicated). As reported in Table 12, R values were generally very low; on this basis, calculated and communicated estimates cannot be classified as comparable nor mutually predictable, [26]. Even more importantly, it should be noted that, even when the exposure and RCR values are calculated using the same tool (i.e., ECETOC TRA) used to estimate the communicated parameters, relevant differences were observed in the obtained exposure and RCR estimates, which are therefore attributable to the differences found between the scenario communicated and that actually observed. However, it should be noted that, if ECETOC TRA is used to calculate the exposure and the RCR values, the uncertainty level is much lower than those defined for the use of the other modeling tools. Despite this, it is also important to note that in some situations the calculated exposure is higher than that the communicated exposure (*N* = 10 using STOFFENMANAGER^®^; *N* = 1 using ART; *N* = 6 using ECETOC TRA (of which *N* = 1 for exposure dermal, *N* = 1 long-term inhalation exposure and *N* = 4 for short-term inhalation exposure). It is of particular interest to note that in the calculation of the short-term inhalation exposure, carried out with ECETOC TRA, the calculated RCR values were in four cases out of four higher than those previously communicated and calculated with the same model (and in three out of four cases this results in a calculated RCR > 1). Further, this occurred in 8 out of 10 cases when using STOFFENMANAGER^®^ (in three cases the calculated RCR was > 1) and in one case out of one for ART (also in this case the calculated RCR > 1) thus highlighting the possibility of encountering “false safe scenarios” [7].

## 4. Discussion

### 4.1. Companies Management of Exposure Scenarios

Before data collection aimed at characterizing the observed ESs, a review was carried out in each of the companies visited regarding the management of those available. By interviewing the managers of the companies and with the help of an external consultant, it was possible to outline a general overview of the difficulties in ESs management in productive realities. In the three companies where the audits were carried out, the same problems were encountered, i.e., (i) communication; (ii) application procedure and (iii) mixtures management.

#### 4.1.1. Communication

Different problems emerged in the communication of the exposure scenarios along the supply chain. Probably, the most important difficulty concerns the problem of obtaining the e-SDSs from the suppliers. The three companies that were visited had the same experience with incomplete, unsupported and outdated ES and no or minimal response from the suppliers, despite a specific REACH provision obliging company to distribute the ES along the supply chain. One of the companies, out of 500 requests, received only 20 ES. Another key point is the translation of the ES in the language of the country where the substance is placed on the market, (i.e., Italian in Italy). In these companies, most of the ES are in English and/or different from Italian. This problem has repercussions on the difficulty of interpretation by experts and employees, especially regarding the activities, RMMs and OCs prepared for safe use. The huge amount of data reported in the ES also leads to a difficult linguistic translation, because the revision requires considerable time by experts and workers. An e-SDS may contain some tenths of ES in a document that can easily have hundreds of pages and for the companies, finding the information that is important can be cumbersome. Communication problems have also been found since the tracking and acknowledgment of the ES, for the manufacturers, importers, suppliers and downstream users, are not always guaranteed. This is due to the lack of efficiency of the management processes, missing of dedicated functionalities in the software and from the reception and sending of SDS and ES in independent formats.

#### 4.1.2. Application Procedure

Regarding the application of the scenarios in the company, it is necessary to check the provisions to be followed upon receipt. Classification and tonnage of purchased substances or mixtures determine different approaches. The main difficulty encountered in the companies was the verification of safe uses. This is summarized in a weak approach due to timing difficulties and lacking experience in this context.

Generic ESs often do not satisfy the specific use of the downstream user with the consequence of the need to perform a scaling process, downstream-user chemical safety report, or technical adjustments, substitutions of substances and/or mixtures and replacement of processes. Many customer companies are also small and medium-sized enterprises, which often do not have the appropriate resources for efficient management. Therefore, checking the customer’s uses is a long and difficult process. Paradoxical situations also occurred in the verification of received SDS and ES, which outlined inconsistencies between communicated ES parameters and the actual (observed) working conditions. This leads to an incorrect interpretation of safe use, RMM and OC, and therefore in the risk for operators in the working activity.

#### 4.1.3. Mixtures Management

Mixture management is another complex problem that occurs in the company’s scenario. The difficulties encountered in the companies are related to the communication and implementation of safe use information for mixtures methodology outlined by top-down and bottom-up approaches [30]. Lead component identification methodology is also difficult to apply as it requires numerous economic and temporal resources to be applied efficiently. This is highly driven by those substances that drive the CLP (classification, labeling and packaging (Regulation (EC) 1272/2008)) classifications of the mixture, the so called “lead components”. The lead component is not necessarily the most hazardous substance in the mixture; other factors need to be considered such as the concentration in the mixture and the exposure route/pathway [30]. Its application concerns the use of numerous parameters including the classification and CLP labeling of the mixture, hazard data (e.g., DNELs), local effects (e.g., irritation, corrosivity and sensitization) and conditions of use affecting exposure (e.g., formation of vapors, dusts, aerosols and use as a solid). This methodology, therefore, concerns priority substances (carcinogens, mutagens and reprotoxic (CMR)), classified substances with DNELs, classified substances with other toxicity reference values, substances that have similar modes of action and biological effect, but differs in the potential to cause combined effects on the basis of concentration and dose. Substances present in the mixture, which can give rise to synergistic effects, additives and/or antagonists are not taken into consideration in this approach. In addition, it is mandatory that the verification of safe uses is carried out both for downstream users and for formulators, therefore it must be consistent with what it is communicated by the suppliers. In this context the contributing activities (PROCs) must be suitably assessed with RMMs and OCs for each substance in the mixture. The evaluation of all these parameters, which is necessary for the application of the methodology, is considerably difficult for companies. This concerns economic resources for properties studies, timing and a lack of dedicated experts. Therefore, the solution adopted is the communication of the ES of each substance that makes up the mixture with the SDS of the mixture itself.

#### 4.1.4. Possible Solutions

Once the general situation regarding the management of the exposure scenarios has been outlined, some measures can be taken for a more aware understanding and management. As far as communication is concerned, the implementation of software capable of guaranteeing the traceability of sending and receiving documents with systems capable of demonstrating their acknowledgment is necessary. In the internal management of the company there must be a procedure for the translation in the appropriate language of the ES. It could also be useful, in the context of the interpretation of the scenarios, to establish a univocal format at European level, also at the level of graphic design. The parameters and phrases, present in SDS and ES, require unique translation into the different European languages. This is to facilitate the interpretation by experts and employers of the productive realities. A significant problem is also related to use verification procedures. Through the selection of specific use descriptors, it is possible in the company to characterize the productive cycle of substances and mixtures. The description of the same is useful for comparing RMM and OC applied and communicated within the received ES. This ensures an informed acquisition of information, which is therefore aimed at implementing the appropriate compliance procedures. The presence of documentation certifying the verification is required and this can be organized easily through the use of specific chemical risk management software, or software that allow to set up customized spreadsheets or databases. Furthermore, the verification of the uses of the downstream user must be carried out through efficient communication. The mixtures management can be optimized through the help of specific guidelines present on dedicated portals of government agencies and sector agencies. Another possibility is to rely on expert consultants, able to outline an efficient strategy aimed at complying with current regulations.

Despite the critical issues that emerged during the audits carried out, the participating companies have proven to be proactive towards the problem by implementing training activities and procedural protocols to optimize the current situation. This shows particular attention to the problem that appears to be topical since the entry into force of the REACH regulation.

### 4.2. Results Discussion

In this study, exposure estimates (calculated via ECETOC TRA) and corresponding RCRs communicated through e-SDS were compared with scenarios studied at actual workplaces (observed scenarios). Exposures in the observed scenarios were calculated using ECETOC TRA, STOFFENMANAGER^®^ and ART. The e-SDS was collected by the authors, from the companies, as well as the contextual information of the exposure scenarios, that was observed onsite. *N* = 25 exposure situations (out of the 49 scenarios reviewed from the e-SDS) were observed, allowing to model the actual exposure situations at the companies and comparing those with the generic scenarios in the registered ES.

The communicated scenarios were not representative of the observed scenarios. Many differences were observed and documented (Table 3 and Table 4), regarding several aspects, including fundamental ones, such as the classification of the PROCs, the duration of the activity, the concentration of the chemical in use, the use and the characteristics of the recommended PPE. The fact that there is a bias between these parameters in the actual exposure scenario and in the communicated scenario, could determine a bias in the exposure estimate and in the calculation of the RCR, as many of the above parameters are descriptors of the estimate used. Further, basic information (e.g., vapor pressure and molecular weight) were preferably collected from the e-SDS for the exposure modeling: however, information about physico-chemical properties, was often missing. These missing data was therefore collected by searching in databases: using faulty data for occupational exposure modeling could have a great impact on exposure calculations and can lead to unreliable estimates.

In this regard, using the same model (ECETOC TRA), the calculated exposure and RCRs had generally lower medians compared to the communicated ones; this is not true when both inhalation short-term exposure and the corresponding RCRs for local effects resulted higher than those communicated, even if the observed differences were not statistically significant. This could be attributed to the fact that the registered RCRs are generic and often refer to worst-case scenarios, while the observed RCRs were based on peculiar scenarios with specific characteristics and contextual information (e.g., type of processing, control measures, etc.) [7].

However, it is troublesome that in 6 out of 40 (15%) exposure situation evaluated with ECETOC TRA, the calculated RCR values were higher than those previously communicated and calculated with the same model and in three out of six cases, the calculated RCR resulted to be greater than 1, (which means ‘false safe’ scenarios). In this regard, the most ‘false safe’ scenarios were detected using STOFFENMANAGER^®^ (*N* = 3 calculated RCR > 1; *N* = 10 calculated RCR > communicated RCR) and the fewest with ART (*N* = 1 RCR > 1). This result appears to be consistent with a previous study [7], which also reports that most of the scenarios with a calculated exposure (and/or RCR) higher than the communicated exposure (and/or RCR), was defined with a Tier 2 model and not with a Tier 1 model. Overall, these results indicate that in some circumstances, the tiered approach for exposure modeling is not working, since Tier 1 models should provide conservative estimates, (and in any case more conservative results than those obtained by Tier 2 models).

Among the Tier 2 models, as already outlined by Landberg et al., [7], one reason STOFFENMANAGER^®^ had many more false safe results than ART in this study could be due to the fact that in STOFFENMANAGER^®^, the uncertainty is included in the estimate used in the calculations, giving higher exposure estimates and possibly resulting in more false safe scenarios; further, it was already discussed that ART may underestimate the exposure in general [31,32], thus giving lower exposure estimates and possibly resulting in less false safe scenarios. In this regard, previous studies also found that ART was he most accurate model among others, even if the model tended to underestimate some particular scenarios and even if the conservatisms of the model defined by the authors as medium, with a tendency of the model to overestimate lower exposure and to underestimate higher exposure [12,33,34,35]. Further, STOFFENMANAGER^®^ may overestimate scenarios with low exposures [32], which may be another one reason for obtaining false safe scenarios for low-DNEL chemicals.

The chemical–physical properties of chemical agents can also play an important role in determining the result of an exposure estimate, potentially leading to errors in overestimation and underestimation. One of these properties is volatility: previous studies outlined that exposure models can assume a different performance in terms of accuracy and precision according to the volatility of the chemical being considered [2,36]. In this regard, it is necessary to note that three of the five chemicals considered have VP <10 Pa (which is considered in this context as a threshold for identifying between volatile and non-volatile substances); however, a significant difference in the comparison of communicated and calculated exposure as a function of the volatility of the considered chemicals was not defined in the present study.

Considering that that vapor pressure was found to be an uncertainty factor for STOFFENMANAGER^®^, and that volatile chemicals will likely result in exposure overestimations [2,12,37], the tendency to observe RCR > 1 using STOFFENMANAGER^®^ could be high for volatile chemicals (especially if combined with a low DNEL) [7]. This leads to the suggestion that generic ES and exposure models should be used with caution (or not at all) when chemicals have both high vapor pressure and low DNELs (i.e., the most harmful chemicals) [7]. Despite these indications, it also should be noted that only few determinants (modifying factors) were deeply evaluated on estimates results. McDonnell and collaborators [32] described scenarios by the means of main modifying factors: (i) activity emission potential, (ii) substance emission potential (categories grouped to dust or granules) and (iii) localized controls. Koivisto and collaborators [38] provided an extensive work on the general ventilation multipliers, which as then further discussed by Cherrie et al. [39], while Park et al. [40] evaluate the ventilation rate, as well as the room size and the amount of aerosol sprayed. Further, two studies performed a sensitivity analysis for investigating modifying factors’ impact on estimates results [12,37]. Therefore, there is no complete information on the role of the different modifying factors or scenario descriptors in determining an impact on the reliability of the models’ estimates.

Similarly, available studies do not currently provide enough information on the reliability of the models for many of the main process categories coded under REACH; [2], therefore, possible errors or observed variations within calculated and communicate exposure estimates could be attributable to limitations of the models’ reliability (i.e., accuracy, precision and robustness), which could be in turn attributable to limitations in their own structure.

Then, as discussed above, it is necessary to make a consideration regarding the reliability of the exposure estimation models. A recent study [2] has reviewed the scientific literature on these aspects and reported the situation regarding the state of the art of the evidence concerning the validation and evaluation studies of the performance of these models. What emerges from this review study is that the available information regarding actual performance of the different models and their effective domain of validity are still scarce. More in detail, studies about the ECETOC TRA outlined that the model was described as not conservative enough to be a tier 1 model in several exposure scenarios, also for the possibility of generating false safe scenarios. Further, some other studies indicated ECETOC TRA’s estimates results should be interpreted carefully, since overestimation or underestimation could be observed as function of the considered scenario. STOFFENMANAGER^®^ resulted to be a balanced and robust model and therefore, the most suitable model to be used in case of uncertainties in the characterization of the scenario. Although a tendency to overestimate low exposures and underestimate high exposures was also observed for STOFFENAMANAGER^®^, which is however sufficiently conservative.

ART is characterized by the tendency to overestimate low exposure levels, but some studies also documented underestimation in some scenarios; despite this, overall the model was found to be conservative and the most accurate and precise. The behavior of the models considered is in line with these general indications, and the results obtained in this study, therefore, are aligned with those observed in previous studies. Precisely in this perspective, despite Tier 1 model and general ES should be the first choice of the industry in assessing the chemical risk, the recommendation Tier 1 models (such as ECETOC TRA) and generic scenarios in the first place appear to be questionable, since results indicate that higher-tier tools (and precisely STOFFENMANAGER^®^), could be considered more protective and identifies more false safe scenarios, when estimates are performed on the actual scenario. Then, STOFFENMANAGER^®^ may be considered as a safer alternative in this context, also considering that, in previous studies, this tool resulted to be robust and the most balanced model within REACH’s Tier 1 and Tier 2 models, and therefore the most suitable model to be used when evaluating exposure scenarios characterized by uncertainty [2].

Again, the obtained evidences, contribute to suggest that the ES assessment should not be performed in a generic manner in absence of sufficient knowledge of the specific environments at all companies [7]; it would be safer for downstream users to perform scenario-specific evaluations, by means of proper modeling tools or with a proper scaling approach, to achieve more representative (and thus safer) estimates of chemical risk.

### 4.3. Limitations and Strengths

The input parameters of the models were reviewed by two external occupational hygienists to avoid low-reliability of assessment due to between-user variability [7,31,41]. Nevertheless, the parameters may differ following the different perspective of the operator and this might introduce mistakes that could lead to miscoding. Anyhow, the possibility to perform a first-person site visit during activities execution is fundamental to obtain good-quality information, thus the onsite observation of the actual exposure scenario was the greatest strength of this study. Further, differences in models estimate can be related to uncertainties related to correlation between model parameters: in this regard a recent study proposed an integrated approach aimed to improve between-user reliability [8]. In this view, TREXMO was suggested as a tool to overcome between-users and between-models biases, but still require further evaluations [2], also in light of some recent implementations [42]. The study was also limited to a small number of considered ESs and exposure situations; thus, although the results obtained are in line with what is defined in other studies—which include a much greater number of ES—it would be thus difficult to generalize the obtained results and draw straightforward conclusions. Therefore, these results, albeit informative and consistent with what has already been defined in other studies, are not suggesting any possible solutions for improving the exposure estimation models used for this study, nor the superiority or inferiority of each model. It should also be noted that for the purposes of this study, environmental monitoring measures have not been carried out: the exposure data measured correctly and instrumentally, when available, is considered as the reference data for the assessment of occupational exposure. The identification of the false safe scenario, with the RCR > 1, was therefore carried out only by means of model estimates but was not subsequently confirmed by means of environmental monitoring measures. The results, however, imply the potential inadequacy of the use of generic scenarios and Tier 1 models in assessing chemical risk in real scenarios of use of chemical agents. The study should be thus repeated with a greater number of ESs to further confirm the obtained results and to further investigate the influence of the possible determinants of the observed differences in exposure estimates, including physico-chemical properties of chemicals, ES characteristics, etc.

## 5. Conclusions

Although the calculated exposure and RCRs generally were lower than communicated (emphasizing a good level of conservatism), the correlation between communicated and calculated exposures and RCRs was generally poor, indicating that the generic communicated scenarios do not reflect actual working, exposure and risk conditions. Further, some observed scenarios resulted in calculated exposure values and RCR higher than those communicated through chemicals’ e-SDSs. Several as ‘false safe’ scenarios (calculated RCRs > 1) were also observed.

Overall, the evidence obtained helps to suggest that the risk assessment cannot be based on the uncritical use of the information provided in generic ES, as these, although generally being conservative, may provide insufficient detail of the specific scenario and may not reflect the actual working, exposure and risk conditions of the scenarios to be evaluated. Contrariwise, evidences suggested that it would be better to perform scenario-specific evaluations, by means of proper use of adequate modeling tools or with a proper scaling approach (i.e., operative processes that allows to recalculate the risk depending on the actual OCs and RMMs associated with the use of the chemical.), to achieve more representative (and thus safer) estimates of chemical risk.

## Figures and Tables

**Table 1:** Chemicals considered in this study, with their chemical abstract service number (CAS No), European community number (EC No) and number of communicated exposure situations ((N)).

Chemical	CAS No.	EC No.	N
Morpholine	110-91-8	203-815-1	11
2,2′-iminodiethanol (90%)	111-42-2	203-868-0	9
3-isocyanatomethyl-3,5,5-trimethylcyclohexyl isocyanate	4098-71-9	223-861-6	13
2,4,6-trichloro-1,3,5-triazine	108-77-0	203-614-9	7
2-[[(butylamino)carbonyl]oxy]ethyl acrylate	63225-53-6	264-036-0	9
Total of considered Exposure Situations			49

**Table 2:** Physico-chemical properties of the chemicals considered in this study and associated reference values for each route of exposure. Note: * = DNEL/DMEL; Sources: GESTIS = substance database (IFA, Berllin, Denmark; www.dguv.de/ifa/gestis-database), PubChem = (https://pubchem.ncbi.nlm.nih.gov/); ECHE = European Chemical Agencies Registered substances factsheets (https://echa.europa.eu/search-for-chemicals); SDS = chemical’s (extended) safety data sheet, as provided by the supplier.

Chemical	Physico-Chemical Properties				Reference Values (DNEL)		
	MW (g/mol)	VP (Pa)	WS (mg/L)	Log Kow	Long-Term-Inhalation Exposure (Systemic Effects) (mg/m^3^)	Long-Term-Inhalation Exposure (Local Effects) (mg/m^3^)	Long-Term-Dermal Exposure (mg/kg/day)
Morpholine	87.12 *(PubChem)*	9.80 × 10^2^ *(ECHA)*	1.0 × 10^6^ *(PubChem)*	−2.55 *(ECHA)*	91 *(ECHA)*	36 *(ECHA)*	1.04 *(ECHA)*
2.2′-iminodiethanol (90%)	105.14 *(PubChem)*	8.55 × 10^−3^ *(ECHA)*	1.0 × 10^6^ *(ECHA)*	−2.00 *(ECHA)*	0.75 *(ECHA)*	0.5 *(ECHA)*	0.13 *(ECHA)*
3-isocyanatomethyl-3.5.5-trimethylcyclohexyl isocyanate	222.29 (GESTIS)	6.30 × 10^-2^ *(ECHA)*	1.5 × 10 *(ECHA)*	4.75 *(SDS)*		0.045 * *(SDS)*	
2.4.6-trichloro-1.3.5-triazine	184.41 *(SDS)*	6.00 × 10^-3^ *(SDS)*	4.4 × 10^2^ *(ECHA)*	2.14 *(SDS)*		0.06 *(SDS)*	6.94 *(SDS)*
2-[[(butylamino)carbonyl]oxy]ethyl acrylate	215.25 *(PubChem)*	1.29 × 10^2^ *(ECHA)*	5.0 × 10^−3^ (SDS)	1.82 *(SDS)*	9.9 *(SDS)*		2.0 *(SDS)*

**Table 3:** Scenarios characteristics represented by scenario name and process categories (PROC) for both the communicated scenario and observed scenario with the number (N) and percentage (%) of each descriptor.

Variable	Communicated Scenario			Observed Scenario		
		*N*	%		*N*	%
Scenario Description	Formulation and packaging/repackaging of substances and mixtures	11	22.4%	Formulation and packaging/repackaging of substances and mixtures	12	38.7%
	Leather adjuvant	9	18.4%	Leather adjuvant	5	16.1%
	Use as an intermediate or monomer	17	34.7%	Use as an intermediate or monomer	6	19.4%
	Loading/filling	3	6.1%	Loading/filling	2	6.5%
	Industrial manufacture of coatings, inks, adhesives and/or other liquid formulations	9	18.4%	Industrial manufacture of coatings, inks, adhesives and/or other liquid formulations	5	16.1%
				Industrial application of coatings, inks, adhesives and/or other formulations	1	3.2%
PROC	PROC1	4	8.2%	PROC3	4	8.2%
	PROC2	5	10.2%	PROC5	3	6.1%
	PROC3	4	8.2%	PROC7	2	4.1%
	PROC4	4	8.2%	PROC9	5	10.2%
	PROC5	2	4.1%	PROC10	3	6.1%
	PROC6	1	2.0%	PROC13	1	2.0%
	PROC7	3	6.1%	PROC15	5	10.2%
	PROC9	4	8.2%	PROC8a	3	6.1%
	PROC10	3	6.1%	PROC8b	5	10.2%
	PROC13	3	6.1%	NOT RELEVANT	18	36.7%
	PROC14	2	4.1%			
	PROC15	4	8.2%			
	PROC8a	4	8.2%			
	PROC8b	6	12.2%			

**Table 4:** Number (N) and percentage (%) of PROC’s characterizing parameters collected for ECETOC TRA v.3.1 in the communicated scenario and observed scenario.

Variable	Communicated Scenario			Observed Scenario	
		*N*	%	*N*	%
Solid	Yes	6	12.2	4	9.3
	No	43	87.8	39	90.7
Dustiness of solids or VP of volatiles at process temperature	Low	1	16.7	0	0
	Medium	5	83.3	4	100
Duration of activity	<15 min	4	8.2	0	0
	15 min to 1 h	4	8.2	16	51.6
	1–4 h	17	34.7	8	25.8
	>4 h	24	49.0	7	22.6
Use of ventilation	Outdoor	1	2.0	0	0
	Indoor	29	59.2	0	0
	Indoor + LEV	16	32.7	23	74.2
	Indoor + LEV + good general ventilation	0	0	7	22.6
	Indoor + LEV + Enhanced General Ventilation	3	6.1	1	3.2
Respiratory protection efficiency	RPE Not in use	33	67.3	0	0
	90%	5	10.2	27	87.1
	95%	9	18.4	4	12.9
	99%	2	4.1	0	0
Substance in preparation	<1%	0	0	25	80.6
	5–25%	20	40.8	2	6.5
	>25%	29	59.2	4	12.9
Dermal PPE/Gloves	APF5	11	22.4	0	0
	APF10	32	65.3	27	87.1
	APF20	6	12.2	4	12.9
LEV (Dermal)	Yes	27	55.1	29	93.5
	No	22	44.9	2	6.5

**Table 5:** Number of valid (*N*) communicated exposure estimates and associated risk characterization ratios (RCRs) for each route of exposure. SD: standard deviation; Min: minimum; Max: maximum.

Communicated Exposures and RCRs	*N*	Mean	SD	Median	Min	Max	Range
Dermal Exposure (mg/kg × day)	36	1.96 × 10^−1^	3.06 × 10^−1^	6.90 × 10^−2^	3.00 × 10^−3^	1.37	1.37
RCR—dermal exposure	33	2.33 × 10^−1^	2.43 × 10^−1^	1.32 × 10^−1^	5.00 × 10^−3^	8.24 × 10^−1^	8.24 × 10^−1^
Inhalation, long-term exposure (systemic effects) (mg/m^3^)	35	3.54	5.79	6.40 × 10^−2^	4.00 × 10^−3^	1.82 × 10	1.82 × 10
RCR—Inhalation, long-term exposure (systemic effects)	24	2.58 × 10^−1^	2.68 × 10^−1^	1.50 × 10^−1^	2.60 × 10^−2^	8.30 × 10^−1^	8.04 × 10^−1^
Inhalation, long-term exposure (local effects) (mg/m^3^)	23	4.31	6.94	2.80 × 10^−2^	4.00 × 10^−3^	1.82 × 10	1.82 × 10
RCR—Inhalation, long-term exposure (local effects)	13	4.21 × 10^−1^	1.77 × 10^−1^	5.11 × 10^−1^	1.54 × 10^−1^	6.13 × 10^−1^	4.59 × 10^−1^
Inhalation, short-term exposure (local effects) (mg/m^3^)	13	2.12 × 10^−2^	8.94 × 10^−3^	2.30 × 10^−2^	7.00 × 10^−3^	3.70 × 10^−2^	3.00 × 10^−2^
RCR—Inhalation, short-term exposure (local effects)	13	4.69 × 10^−1^	1.96 × 10^−1^	5.11 × 10^−1^	1.54 × 10^−1^	8.18 × 10^−1^	6.64 × 10^−1^

**Table 6:** Number of valid (N) exposure estimates calculated with ECETOC TRA v.3.1 model and associated RCRs for each route of exposure evaluated. SD: standard deviation; Min: minimum; Max: maximum.

Calculated Exposure and RCR Tool: ECETOC TRA v.3.1	*N*	Mean	SD	Median	Min	Max	Range
Dermal Exposure (mg/kg × day)	31	8.44 × 10^−2^	2.96 × 10^−1^	6.90 × 10^−3^	3.00 × 10^−4^	1.65	1.65
RCR—Dermal exposure	27	1.66 × 10^−1^	4.33 × 10^−1^	2.06 × 10^−2^	4.90 × 10^−5^	2.11	2.11
Inhalation, long-term exposure (systemic effects) (mg/m^3^)	31	7.72 × 10^−2^	1.97 × 10^−1^	2.69 × 10^−2^	3.50 × 10^−5^	1.09	1.09
RCR—Inhalation, long-term exposure (systemic effects)	31	4.20 × 10^−2^	1.33 × 10^−1^	3.50 × 10^−3^	1.00 × 10^−4^	7.20 × 10^−1^	7.20 × 10^−1^
Inhalation, short-term exposure (local effects) (mg/m^3^)	31	6.31 × 10^−1^	9.58 × 10^−1^	1.45 × 10^−1^	2.00 × 10^−4^	4.36	4.36
RCR—Inhalation, short-term exposure (local effects)	25	6.02 × 10^−1^	1.07	2.02 × 10^−2^	4.00 × 10^−4^	3.70	3.70

**Table 7:** Number of valid (N) exposure estimates calculated with STOFFENMANAGER^®^ v.8.0 model and associated RCRs for each route of exposure evaluated. SD: standard deviation; Min: minimum; Max: maximum.

Calculated Exposure and RCR Tool: STOFFENMANAGER^®^ v.8.0	*N*	Mean	SD	Median	Min	Max	Range
Inhalation, long-term exposure (systemic effects) (mg/m^3^)	31	2.45	5.91	4.0 × 10^−1^	1.70 × 10^−4^	3.25 × 10	3.25 × 10
RCR—Inhalation, long-term exposure (systemic effects)	31	2.91	1.09 × 10	3.10 × 10^−2^	2.00 × 10^−3^	5.67 × 10	5.67 × 10
Inhalation, short-term exposure (local effects) (mg/m^3^)	25	2.00	6.45	6.00 × 10^−2^	1.70 × 10^−4^	3.25 × 10	3.25 × 10
RCR—Inhalation, short-term exposure (local effects)	25	3.54	1.21 × 10	3.20 × 10^−2^	2.80 × 10^−3^	5.67 × 10	5.67 × 10

**Table 8:** Number of valid (N) exposure estimates calculated with ART v.1.5 model and associated RCRs for each route of exposure evaluated. SD: standard deviation; Min: minimum; Max: maximum.

Calculated Exposure and RCR Tool: ART v.1.5	*N*	Mean	SD	Median	Min	Max	Range
Inhalation, long-term exposure (systemic effects) (mg/m^3^)	23	3.67	1.49 × 10	3.00 × 10^−2^	2.30 × 10^−4^	7.20 × 10	7.20 × 10
RCR—Inhalation, long-term exposure (systemic effects)	23	1.14 × 10^−1^	3.37 × 10^−1^	3.00 × 10^−3^	3.10 × 10^−4^	1.47	1.47
Inhalation, short-term exposure (local effects) (mg/m^3^)	24	3.99	1.47 × 10	5.00 × 10^−3^	3.50 × 10^−4^	7.20 × 10	7.20 × 10
RCR—Inhalation, short-term exposure (local effects)	24	1.19 × 10	5.33 × 10	4.00 × 10^−3^	4.6 × 10^−5^	2.67 × 10^2^	2.67 × 10^2^

**Table 9:** Comparison and difference between communicated exposure estimates and calculated exposure estimates with ECETOC TRA v.3.1 with associated RCRs. N: number of valid exposure estimates. SD: standard deviation.

Communicated	*N*	Mean	Median	SD	Range
Dermal Exposure (mg/kg × day)	21	2.24 × 10^−1^	6.90 × 10^−2^	3.53 × 10^−1^	1.37
RCR—Dermal exposure	21	2.35 × 10^−1^	1.32 × 10^−1^	2.40 × 10^−1^	8.20 × 10^−1^
Inhalation, long-term exposure (systemic effects) (mg/m^3^)	20	3.35	7.04 × 10^−1^	5.58	1.81 × 10
RCR—Inhalation, long-term exposure (systemic effects)	20	2.39 × 10^−1^	1.36 × 10^−1^	2.54 × 10^−1^	8.04 × 10^−1^
Inhalation, short-term exposure (local effects) (mg/m^3^)	4	1.78 × 10^−2^	1.80 × 10^−2^	9.50 × 10^−3^	2.10 × 10^−2^
RCR—Inhalation, short-term exposure (local effects)	4	0.39	0.4	0.21	0.46
**Calculated (ECETOC TRA v.3.1)**	***N***	**Mean**	**Median**	**SD**	**Range**
Dermal Exposure (mg/kg × day)	25	3.19 × 10^−2^	6.90 × 10^−3^	6.29 × 10^−2^	2.74 × 10^−1^
RCR—Dermal exposure	25	1.38 × 10^−1^	2.06 × 10^−2^	4.55 × 10^−1^	2.11
Inhalation, long-term exposure (systemic effects) (mg/m^3^)	25	4.63 × 10^−2^	2.69 × 10^−2^	6.53 × 10^−2^	2.69 × 10^−1^
RCR—Inhalation, long-term exposure (systemic effects)	25	4.87 × 10^−2^	2.70 × 10^−3^	1.47 × 10^−1^	7.20 × 10^−1^
Inhalation, short-term exposure (local effects) (mg/m^3^)	25	4.92 × 10^−1^	1.45 × 10^−1^	6.35 × 10^−1^	2.15
RCR—Inhalation, short-term exposure (local effects)	25	6.02 × 10^−1^	2.18 × 10^−2^	1.18	3.70
**Communicated—Calculated (ECETOC TRA v.3.1) difference**	***N***	**Mean**	**Median**	**SD**	**Range**
Dermal Exposure (mg/kg × day)	21	1.90 × 10^−1^	4.00 × 10^−2^	3.60 × 10^−1^	1.57
RCR—Dermal exposure	21	1.00 × 10^−1^	1.00 × 10^−1^	4.30 × 10^−1^	2.36
Inhalation, long-term exposure (systemic effects) (mg/m^3^)	20	3.29	6.90 × 10^−1^	5.55	1.80 × 10
RCR—Inhalation, long-term exposure (systemic effects)	20	2.30 × 10^−1^	1.30 × 10^−1^	2.50 × 10^−1^	8.40 × 10^−1^
Inhalation, short-term exposure (local effects) (mg/m^3^)	4	−9.00 × 10^−2^	−1.10 × 10^−1^	6.00 × 10^−2^	1.40 × 10^−1^
RCR—Inhalation, short-term exposure (local effects)	4	−2.05	−2.55	1.39	3.08

**Table 10:** Comparison and difference between communicated exposure estimates and calculated exposure estimates with STOFFENMANAGER^®^ v.8.0 with associated RCRs. N: number of valid exposure estimates. SD: standard deviation.

Communicated	*N*	Mean	Median	SD	Range
Inhalation. Long-term exposure (systemic effects) (mg/m^3^)	20	3.35	7.04 × 10^−1^	5.58	1.81 × 10
RCR—Inhalation. Long-term exposure (systemic effects)	20	2.39 × 10^−1^	1.36 × 10^−1^	2.54 × 10^−1^	8.04 × 10^−1^
Inhalation. Long-term exposure (local effects) (mg/m^3^)	9	5.76	2.72	7.61	1.81 × 10
RCR—Inhalation. Long-term exposure (local effects)	9	3.94 × 10^−1^	4.04 × 10^−1^	2.07 × 10^−1^	4.59 × 10^−1^
**Calculated (STOFFENMANAGER^®^ v.8.0)**	***N***	**Mean**	**Median**	**SD**	**Range**
Inhalation. Long-term exposure (systemic effects) (mg/m^3^)	25	2.67	4.00 × 10^−1^	6.54	3.25 × 10
RCR—Inhalation. Long-term exposure (systemic effects)	25	3.57	3.10 × 10^−2^	1.21 × 10	5.67 × 10
Inhalation. Long-term exposure (local effects) (mg/m^3^)	20	2.20	3.80 × 10^−2^	7.19	3.25 × 10
RCR—Inhalation. Long-term exposure (local effects)	20	4.38	3.04 × 10^−2^	1.35 × 10	5.67 × 10
**Communicated—Calculated (STOFFENMANAGER^®^ v.8.0) difference**	***N***	**Mean**	**Median**	**SD**	**Range**
Inhalation. Long-term exposure (systemic effects) (mg/m^3^)	20	0.07	7.75	0.02	40.84
RCR—Inhalation. Long-term exposure (systemic effects)	20	−5.66	15.4	−0.14	56.74
Inhalation. Long-term exposure (local effects) (mg/m^3^)	9	1.59	11.6	0.02	40.84
RCR—Inhalation. Long-term exposure (local effects)	9	0.3	0.13	0.31	0.32

**Table 11:** Comparison and difference between communicated exposure estimates and calculated exposure estimates with ART v.1.5 with associated RCRs. N: number of valid exposure estimates. SD: standard deviation.

Communicated	*N*	Mean	Median	SD	Range
Inhalation, long-term exposure (systemic effects) (mg/m^3^)	20	3.35	7.04 × 10^−1^	5.58	1.81 × 10
RCR—Inhalation, long-term exposure (systemic effects)	20	2.39 × 10^−1^	1.36 × 10^−1^	2.54 × 10^−1^	8.04 × 10^−1^
Inhalation, long-term exposure (local effects) (mg/m^3^)	9	5.76	2.72	7.61	1.81 × 10
RCR—Inhalation, long-term exposure (local effects)	9	3.94 × 10^−1^	4.04 × 10^−1^	2.07 × 10^−1^	4.59 × 10^−1^
**Calculated (ART v.1.5)**	***N***	**Mean**	**Median**	**SD**	**Range**
Inhalation. Long-term exposure (systemic effects) (mg/m^3^)	17	6.18 × 10^−1^	3.00 × 10^−2^	1.47	5.70
RCR—Inhalation. Long-term exposure (systemic effects)	17	1.56 × 10^−2^	3.03 × 10^−3^	2.73 × 10^−2^	1.00 × 10^−1^
Inhalation. Long-term exposure (local effects) (mg/m^3^)	19	1.20	4.10 × 10^−3^	2.98	1.20 × 10
RCR—Inhalation. Long-term exposure (local effects)	19	1.55 × 10	4.60 × 10^−3^	6.11 × 10	2.67 × 10^2^
**Communicated–Calculated (ART v.1.5) difference**	***N***	**Mean**	**Median**	**SD**	**Range**
Inhalation. Long-term exposure (systemic effects) (mg/m^3^)	16	3.52	5.82	1.51	17.93
RCR—Inhalation. Long-term exposure (systemic effects)	16	0.14	0.16	0.05	0.5
Inhalation. Long-term exposure (local effects) (mg/m^3^)	8	3.84	9.9	1.7	29.93
RCR—Inhalation. Long-term exposure (local effects)	8	−66.27	133.19	0.22	266.56

**Table 12:** Regression parameters between communicated and calculated exposure and RCR. The communicated exposure estimates and RCRs were used as the independent variables and the calculated exposure estimates and RCRs were used as the dependent variable in the linear regression analysis.

Communicated * vs. Calculated Exposure	Tool	*N*	Slope			Intercept			Pearson Correlation		Uncertainty	*N* Underestimated Exposure
			m	SE	*p*	q	SE	*p*	R	*p*		
Dermal Exposure (Mg/kg × day)	ECETOC TRA	21	−0.007	0.044	0.877	0.039	0.18	0.044	0.036	0.439	0.08	1
Inhalation Long-term exposure (systemic effects) (mg/m^3^)	ECETOC TRA	20	0.006	0.003	0.053	0.035	0.017	0.054	0.438	0.027	20.0	1
	STOFFENMANAGER^®^	20	0.371	0.292	0.220	2.034	1.863	0.289	0.287	0.11	28.6	9
	ART	16	0.058	0.066	0.390	0.412	0.469	0.395	0.231	0.194	22.0	0
Inhalation Long-term exposure (local effects) (mg/m^3^)	STOFFENMANAGER^®^	9	0.32	0.515	0.555	2.325	4.739	0.6399	0.228	0.277	61.0	1
	ART	8	−0.153	0.215	0.502	3.631	2.098	0.134	0.28	0.251	50.2	1
Inhalation Long-term exposure (local effects) (mg/m^3^)	ECETOC TRA	4	3.432	4.305	0.509	0.049	0.084	0.619	0.491	0.254	0.006	4
Communicated * vs. Calculated RCR	**Tool**	***N***	**Slope**			**Intercept**			**Pearson correlation**		**Uncertainty**	***N* RCR_calc_ > RCR_com_ (false safe)**
			**m**	**SE**	***p***	**q**	**SE**	***p***	**R**	***p***		
Dermal Exposure	ECETOC TRA	21	0.199	0.112	0.091	0.208	0.052	0.001	0.378	0.046	0.09	1 (0)
Inhalation Long-term exposure (systemic effects)	ECETOC TRA	20	0.371	0.292	0.22	2.034	1.863	0.289	−0.007	0.49	0.06	1 (0)
	STOFFENMANAGER^®^	20	2.102	16.828	0.903	5.399	5.761	0.366	0.035	0.451	127	8 (3)
	ART	16	0.07	0.054	0.225	0.004	0.012	0.737	0.398	0.113	0.02	0 (0)
Inhalation Long-term exposure (local effects)	STOFFENMANAGER^®^	9	0.392	0.148	0.118	−0.061	0.064	0.44	0.882	0.059	0.05	0 (0)
	ART	8	−0.153	0.215	0.502	3.631	2.098	0.134	0.672	0.265	8848	1 (1)
Inhalation Long-term exposure (local effects)	ECETOC TRA	4	3.349	4.443	0.53	1.126	1.921	0.586	0.47	0.265	2.83	4 (3)

Legend: N: number of data; R: Pearson correlation coefficient; p: significance; m: slope; q: intercept; SE: standard error. N underestimated exposure: number of situations in which calculated exposure > communicated exposure. N RCRcalc > RCRcom: number of situations in which calculated RCR > communicated RCR; (false safe): number of situations in which calculated RCR > 1. * Communicated exposure values and RCR were obtained through ECETOC TRA.

## Abbreviations

APF: Assigned Protection Factor; ART: Advanced REACH Tool; ECETOC-TRA: European Centre for Ecotoxicology and Toxicology Of Chemicals; Targeted Risk Assessment; ECHA: European Chemical Agency; ES: Exposure Scenario; e-SDS: Extended Safety Data Sheet; LEV: Local Exhaust Ventilation; OC: Operating Conditions; PPE: Personal Protective Equipment; PROC: Process Categories; RCR: Risk Characterization Ratio; REACH: Registration, Evaluation, Authorization of Chemicals (Regulation (EC) 1907/2006); RMMs: Risk Management Measures; RPE: Respiratory Protective Equipment; SDS: Safety Data Sheet.
